# AGG interruptions and maternal age affect *FMR1* CGG repeat allele stability during transmission

**DOI:** 10.1186/1866-1955-6-24

**Published:** 2014-07-30

**Authors:** Carolyn M Yrigollen, Loreto Martorell, Blythe Durbin-Johnson, Montserrat Naudo, Jordi Genoves, Alessandra Murgia, Roberta Polli, Lili Zhou, Deborah Barbouth, Abigail Rupchock, Brenda Finucane, Gary J Latham, Andrew Hadd, Elizabeth Berry-Kravis, Flora Tassone

**Affiliations:** 1 Department of Biochemistry and Molecular Medicine, University of California, Davis, School of Medicine, 2700 Stockton Blvd., Suite 2102, Sacramento, CA 95817, USA; 2 Molecular Genetics Section, Hospital Sant Joan de Déu, Barcelona, Spain; 3 Department of Public Health Sciences, University of California, Davis, Davis, CA, USA; 4 Laboratory of Molecular Genetics of Neurodevelopment, Department of Women’s and Children’s Health, University of Padova, Padova, Italy; 5 Department of Pediatrics, Neurological Sciences, and Biochemistry, Rush University Medical Center, Chicago, IL, USA; 6 Dr. John T. Macdonald Foundation, Department of Human Genetics, Miller School of Medicine, University of Miami, Miami, FL, USA; 7 Geisinger Autism and Developmental Medicine Institute, Lewisburg, PA, USA; 8 Asuragen, Inc., Austin, TX, USA; 9 MIND Institute, University of California, Davis, School of Medicine, Davis, CA, USA

**Keywords:** AGG interruptions, premutation, *FMR1*, gray/intermediate allele, full mutation, risk of expansion

## Abstract

**Background:**

The presence of AGG interruptions in the CGG repeat locus of the fragile X mental retardation 1 (*FMR1*) gene decreases the instability of the allele during transmission from parent to child, and decreases the risk of expansion of a premutation allele to a full mutation allele (the predominant cause of fragile X syndrome) during maternal transmission.

**Methods:**

To strengthen recent findings on the utility of AGG interruptions in predicting instability or expansion to a full mutation of *FMR1* CGG repeat alleles, we assessed the outcomes of 108 intermediate (also named gray zone) and 710 premutation alleles that were transmitted from parent to child, and collected from four international clinical sites. We have used the results to revise our initial model that predicted the risk of a maternal premutation allele expanding to a full mutation during transmission and to test the effect of AGG interruptions on the magnitude of expanded allele instability of intermediate or premutation alleles that did not expand to a full mutation.

**Results:**

Consistent with previous studies, the number of AGG triplets that interrupts the CGG repeat locus was found to influence the risk of allele instability, including expansion to a full mutation. The total length of the CGG repeat allele remains the best predictor of instability or expansion to a full mutation, but the number of AGG interruptions and, to a much lesser degree, maternal age are also factors when considering the risk of transmission of the premutation allele to a full mutation.

**Conclusions:**

Our findings demonstrate that a model with total CGG length, number of AGG interruptions, and maternal age is recommended for calculating the risk of expansion to a full mutation during maternal transmission. Taken together, the results of this study provide relevant information for the genetic counseling of female premutation carriers, and improve the current predictive models which calculate risk of expansion to a full mutation using only total CGG repeat length.

## Background

Soon after the discovery that expansion of the CGG trinucleotide repeat in the fragile X mental retardation 1 (*FMR1*) gene was the causative mutation of fragile X syndrome (FXS) [1-3], AGG triplets interspersed within the *FMR1* allele were observed and hypothesized to stabilize the gene during transmission [4-8] by decreasing the risk of DNA polymerase slippage during DNA replication [9].

Studies that have investigated the role of AGG interruptions in *FMR1* gene expansions have converged on a number of important conclusions: (1) AGG interruptions influence the stability of the CGG repeats within the *FMR1* gene during parental transmission [10,11]; (2) the presence or absence of AGG interruptions was not correlated with transcriptional or translational activity of the gene [12-15]; and (3) AGG interruption patterns can vary greatly between populations, but are for the most part, inherited without change [4,16].

Using a PCR amplification technique that determines the AGG interruption number and position on both maternal alleles [17-19], recent studies have demonstrated that AGG interruptions alter the frequency of genetic instability of intermediate and premutation sized alleles during transmission from parent to child. In 2012, Yrigollen et al. [10] reported that alleles with the same CGG repeat number had a lower risk of full mutation expansion when AGG interruptions were present. The risk inversely correlated with an increase in the number of AGG interruptions within the CGG repeat tract. This study also suggested that maternal age might contribute to the risk of the allele expanding to the full mutation, but the results were not statistically significant. Last, the study proposed a model that incorporated both the AGG interruption status and total CGG length data to predict risk of expansion to a full mutation.

A more recently published, large multicenter study of 457 transmissions from maternal alleles with 45 to 69 repeats also demonstrated that AGG interruptions significantly increase the stability of intermediate and small premutation sized alleles during parental transmission [11]. Indeed, all nine of the full mutation expansions in this study were limited to mothers lacking AGG interruptions [11]. The authors further showed that the magnitude of change in allele size during an unstable transmission inversely correlated with the number of AGG interruptions. Finally, inclusion of the AGG interruption status in a prediction model (as uninterrupted 3′ repeat length) improved the prediction of transmission instability by increasing the proportion of explained variance by 1.9-fold compared to repeat length alone [11].

AGG interruptions information was recently used in a study looking at instability of premutation alleles in a five-generation family identified during a pilot study of newborn screening for FXS, looking therefore to individuals from the general population [20,21]. The presence of AGG interruptions in premutation alleles from 12 individuals (14 transmissions) within the family was observed and a large percentage of unstable (defined by +1 CGG or more) transmissions were seen, but no expansions to a full mutation, consistent with recent reports [10,11].

To extend previous results that the presence of AGG interruptions decreases the risk of an allele expanding to a full mutation, and guide the selection of the most informative model for risk estimates, we analyzed 342 transmissions from 261 mothers with a premutation allele. These transmissions were also combined with the 368 transmissions (out of 373 which included five alleles that decreased in size and that were therefore not included in this study) from 264 mothers, previously published [10], to construct an updated risk prediction model. These newly acquired transmissions were not additional children of the original 264 mothers. The larger transmission dataset was also used to test whether maternal age contributes to the risk of a premutation allele expanding to a full mutation.

This study also analyzed 244 transmissions that did not result in a full mutation, with the inclusion of both intermediate (62 mothers and 13 fathers with 45 to 54 CGG repeats) and small premutation (117 mothers with 55 to 100 CGG repeats) alleles, to determine how AGG interruptions within these alleles affected the magnitude of repeat size instability that was observed.

Here we present our results on the risk of expansion to a full mutation in 710 transmissions from 525 premutation carrier mothers, and magnitude of instability in 244 transmissions that did not expand to a full mutation from 75 intermediate and 117 premutation carrier parents (Additional file 1: Table S1).

The combined dataset, which represents the largest cohort studied so far for risk of expansion to a full mutation during maternal transmission, provides valuable insights for genetic counseling of individuals with expanded alleles. In particular, knowledge of AGG interruptions within the CGG repeats contributes to improve the predictive risk estimates for expansion of a premutation allele to a full mutation in the subsequent generation.

## Methods

### Participants

Individuals were recruited through the Fragile X Research and Treatment Center at the MIND Institute - University of California Davis, USA; the Molecular Genetics Department of the Hospital Sant Joan de Déu, Barcelona, Spain; the Pediatric Neurology Unit and Rare Disease Laboratory of the University of Padova, Italy; and Rush University Medical School, Chicago, Illinois, USA. Whole blood was collected following informed consent and according to protocols approved by the respective Institutional Review Boards. The inclusion criteria were women with intermediate and premutation alleles and children who inherited the expanded allele and men with intermediate alleles plus their offspring who inherited the expanded allele. Samples that contracted in size were excluded when predicting the risk of expansion to a full mutation, as the mechanisms that are involved in contraction are likely different than those involved in expansion of the CGG repeat allele [22]. The participants were selected based on previously performed DNA molecular testing for intermediate, premutation, or full mutation alleles by southern blot and PCR analyses [18] at one of the above mentioned testing centers who were enrolled in different ongoing research studies in the three sites or were seen clinically because of a family history of FXS.

Expansion was defined as an allele that expanded from a premutation to a full mutation. Magnitude of expansion was defined as an allele (intermediate or premutation) increasing in size of greater than 1 CGG repeats (that did not go into a full mutation allele).

### Molecular characterization

To determine the structure of the CGG repeat element of the *FMR1* gene, each data collection site (University of California Davis, Hospital Sant Joan de Déu, University of Padova, and Rush University) performed a three primer PCR protocol followed by PCR fragment analysis by capillary electrophoresis. Detailed descriptions of the PCR, which includes a CGG primer, and fragment analysis have previously been reported [17-19]. PCR products were separated and detected by capillary electrophoresis according to manufacturer recommendations. The length of the *FMR1* CGG repeat element, number of AGG interruptions, and position of AGG interruptions were determined (by PCR through both sides when necessary) by analysis of the electropherograms using size markers spanning the entire PCR range [10].

### Statistical analysis

The transmission results from premutation carrier mothers were analyzed to determine if an association between AGG interruptions and transmission outcome was present using mixed effects logistic regression models, with total CGG length, pure CGG stretch, maternal age, and collection site as fixed effects and mother as a random effect. Analyses were also performed to test if maternal age (evaluated as a continuous variable) influenced the risk of an allele expanding to a full mutation during maternal transmission.

A model that predicted the risk of expansion to a full mutation given total CGG length and number of AGG interruptions was generated using the logistic regression analysis.

Akaike Information Criterion (AIC) determined the best model for risk of a full mutation. Models that considered one or more variables (total CGG length, pure CGG stretch, maternal age, and number of AGG interruptions) were combined in numerous arrangements and an AIC score was assigned. A lower AIC score is indicative of a better model.

Linear mixed effects models for parent were used to analyze the magnitude of expansion as a function of total CGG length, AGG interruptions, sex of the parent, and parental age. Binomial logistic regression models were used to model the probability of instability as a function of the same covariates. The transmission results were analyzed with maternal and paternal transmissions both separated and joined together; data from the collection sites were included in the model as covariates.

### AGG distribution pattern within normal alleles

We compared the AGG distribution pattern of 514 normal range alleles from premutation carrier women to 797 normal range alleles from women with two normal range alleles. Comparisons of 367, 58, and 89 normal alleles from premutation mothers (from the three sites) were made to 209, 428, and 160 normal alleles screened from women from the general population (UC Davis, Rush University, and Spain, respectively). The distribution of AGG interruption patterns in normal alleles was compared between normal and premutation subjects, stratified by site, using Cochran-Mantel-Haenszel tests. The total CGG length was compared between normal and premutation subjects, adjusting for site, using a multiple linear regression model. The distribution of AGG interruption patterns was compared between normal and premutation subjects at each site using chi-square tests. Data from the Padova site were excluded from the analysis, as no data from normal subjects were available from this site.

Analyses were conducted using R, version 3.0.2 [23].

## Results

### Risk of expansion to a full mutation during maternal transmission

**Figure 1:**
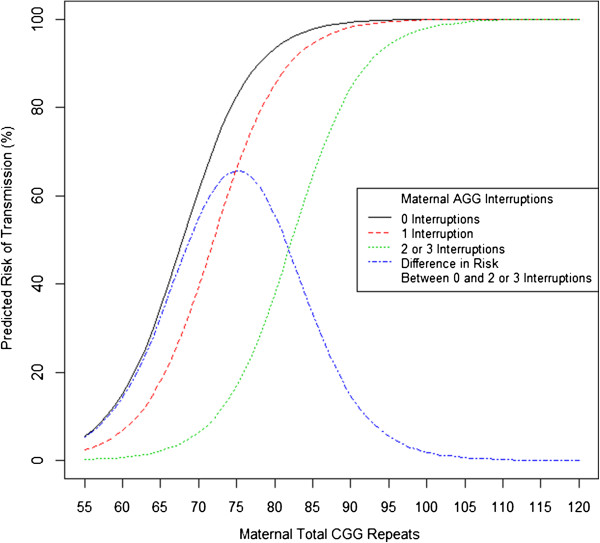
**Predicted risk of a maternal premutation allele expanding to a full mutation during transmission.** Risk calculated using total CGG length and separately for 0, 1, and 2 or 3 AGG interruptions (black, red, and green lines, respectively) in 710 transmissions. The difference in the predicted risk of expansion to a full mutation between alleles with 0 and 2 or 3 AGG interruptions is shown as a blue line.

The reported predicted risks of a premutation allele expanding to a full mutation allele during maternal transmission are based on the analyses of 710 transmission events from 525 premutation carrier mothers using CGG total length and number of AGG interruptions as variables (Figure 1, Additional file 1: Table S2). A summary of the transmissions observed is provided in Additional file 1: Table S3.

**Figure 2:**
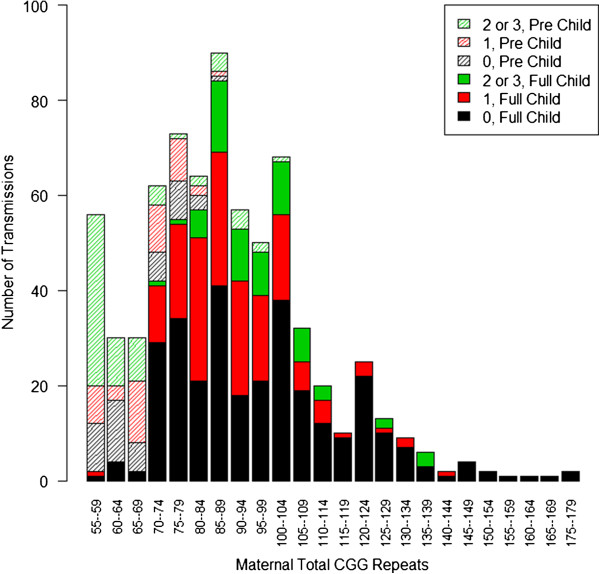
**Distribution of premutation allele total length.** A histogram of the number of transmissions observed for each total CGG repeat length, grouped by 0 (black), 1 (red), and 2 or 3 (green) AGG interruptions. Transmissions that resulted in a premutation are partially shaded, and transmissions that expanded to a full mutation are shown as solid bars.

A model of the predicted risk of expansion to a full mutation using total CGG repeat length and number of AGG interruptions as risk factors showed no significant difference in any of the three sites analyzed separately compared to the combined data (135 UC Davis, 86 Rush University, and 105 Hospital Sant Joan de Dèu). University of Padova was not analyzed separately due to the smaller sample size, but was included in the analyses that combined sites together. In each of the groups the frequency of expansion to a full mutation increased with the total length of the CGG repeat. An overall lower frequency was observed in alleles between approximately 60 and 80 total CGG repeats when the allele contained 1 AGG interruption compared to 0, and with 2 or 3 AGG interruptions when compared to 1 or 0. A model of the predicted risk of expansion to a full mutation using total CGG repeat length and number of AGG interruptions as risk factors showed no significant difference in any of the three sites analyzed separately compared to the combined data. These findings also indicate that expansion risk is not related to ethnicity as the ethnic composition is different in the three sites being as follows: UC Davis: 64% White, 7% Black, 38% Hispanic; Rush University: 22% White, 38% Black, 29% Hispanic; Hospital Sant Joan de Dèu: approximately 100% Hispanic.The transmission results across sites were combined and analyzed. The majority of transmissions were between 70 and 100 repeats. The distribution of maternal total CGG length for the 710 transmissions is shown in Figure 2.

An increase in frequency of expansion to a full mutation was observed in alleles with fewer AGG interruptions, with the most frequent expansions in alleles with 0 AGG interruptions, and the least frequent with alleles that contained 2 or 3 AGG interruptions. The smallest maternal allele to expand to a full mutation was 56 CGG repeats and lacked AGG interruptions. All alleles with greater than 110 CGG repeats expanded to a full mutation (Additional file 2: Figure S1).

The results of a logistic regression model of risk of transmission by total length and AGG interruption shows that the odds of transmission to a full mutation increase significantly with total length (*P* <0.001). For each additional CGG repeat there is an estimated 25% increase in the odds ratio for expansion to a full mutation; as such, a subject with 0 AGG interruptions and a total length of 75 repeats would have an odds ratio 4.7 to 1 of having a full mutation expansion (that is, a probability of 0.83) and a subject with 0 AGG interruptions and 76 repeats would have an odds ratio of 5.9 to 1 (that is, a probability of 0.85). Conversely, the odds decrease significantly with increasing numbers of AGG interruptions, equal to 2.4-fold with a *P* = 0.013 for 1 interruption *vs*. 0 interruptions and, to 23-fold with a *P* <0.001 for 2 or 3 *vs*. 0 interruptions.

A logistic regression model of risk of transmission to a full mutation by total length, AGG interruptions, and maternal age of the 531 cases for which maternal age was available shows an additive effect between maternal age and instability of the allele. Age was still significant in a model with only total length and age (not including number of AGG interruptions) (*P* = 0.011 for age effect) and only pure stretch and age (*P* = 0.001 for age effect). Total length and AGG interruptions similarly affected risk in this analysis compared to the model without age. The odds of expansion to a full mutation during maternal transmission were predicted to increase for each additional year of age (*P* = 0.001) suggestive of an additive effect between maternal age and instability of the allele. To illustrate, the predicted probability of expansion of 56% (that is, an odds of 1.3 to 1) in a 20-year-old mother would increase to 85% (that is, an odds of 5.6 to 1) for a mother aged 30 years (an 16% increase in the odds each year).

### Risk model

**Figure 3:**
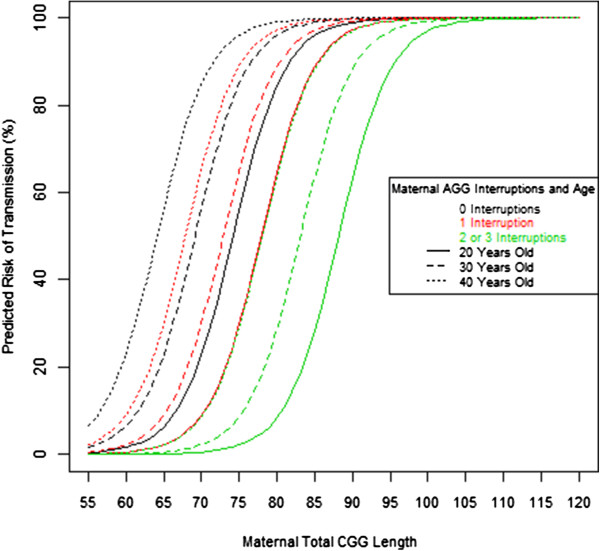
**Maternal age, total length, and number of AGG predict risk of full mutation expansion.** The diagram shows the risk of expansion to a full mutation in a 20-, 30-, and 40-year-old premutation carrier mother predicted for 0 (black), 1 (red), and 2 or 3 (green) AGG interruptions and total length of CGG repeats.

The AIC score for total length and number of AGG interruptions using 710 transmissions was found to be 252.8 and decreased to 241.6 when maternal age was included. Total CGG length, pure CGG stretch, number of AGG interruptions, and maternal age were all tested within 1, 2, or 3 variable models and given an AIC score that measures the fit of the model to the observed data and the number of variables within the model. The scores ranged from 235.6 (a model with pure stretch, AGG number, and maternal age as variables) to 444.8 (a model using only maternal age). The AIC for length of the pure stretch and number of AGG interruptions was 245.7 and decreased to 235.6 when maternal age was included. A comparison of the two primary models (one that includes total length, number of AGG interruptions, and maternal age and another that considers pure stretch, number of AGG interruptions and maternal age) indicated that the model that included total length rather than pure stretch produced risk estimates that were more consistent with our findings regarding the effect each variable had on risk. The model with the lowest AIC (pure stretch, number of AGG interruptions, and maternal age) predicted the highest risk of expansion to a full mutation to occur in alleles with 1 AGG interruption, a deviation from expected outcomes. This deviation is likely the result of insufficient observations of 2 or 3 AGG interruption transmissions. The predictive risk model calculated using total CGG length, number of AGG interruptions, and maternal age is illustrated in Figure 3. Table 1 summarizes the predicted risks of expansion to a full mutation of a premutation carrier female at 20, 30, and 40 years of age at the time of child birth. Noticeably, a differentiated risk of 94% is observed between a 20-year-old mother with 2 AGG interruptions (lower risk group) and a 40-year-old mother with 0 AGG interruptions (higher risk group) for an allele with a total length of 75 CGG repeats. Maternal age was compared for transmissions that resulted in a premutation to those that resulted in a full mutation, to test for ascertainment bias. No significant age difference was found (*P* = 0.975), with the mean age in both groups being 29.7 years.

**Table 1:** Risk of full mutation expansion using total CGG length, AGG, age from 525 premutation mothers

**Total length**	**Age (years)**	**0 AGG**	**1 AGG**	**2 or 3 AGG**	**Differential risk**^**a**^
**55**	20	0.4% (0.1%, 2.3%)	0.1% (0%, 0.9%)	0% (0%, 0.1%)	0.40%
	30	1.6% (0.4%, 6.6%)	0.5% (0.1%, 2.6%)	0% (0%, 0.3%)	1.60%
	40	6.5% (1.5%, 24.3%)	2.3% (0.5%, 9.9%)	0.1% (0%, 1.2%)	6.40%
**60**	20	1.5% (0.3%, 7.3%)	0.5% (0.1%, 2.9%)	0% (0%, 0.3%)	1.50%
	30	6.4% (2%, 18.8%)	2.2% (0.6%, 7.7%)	0.1% (0%, 0.9%)	6.30%
	40	23.1% (7.3%, 53.5%)	9.1% (2.6%, 27.4%)	0.5% (0.1%, 3.7%)	22.60%
**65**	20	6.3% (1.7%, 21.2%)	2.2% (0.5%, 9%)	0.1% (0%, 0.9%)	6.20%
	30	22.9% (9.8%, 44.7%)	9% (3.4%, 21.6%)	0.5% (0.1%, 2.7%)	22.40%
	40	56.6% (27.4%, 81.8%)	30.2% (12%, 58%)	2.1% (0.4%, 11.5%)	54.50%
**70**	20	22.7% (8%, 49.7%)	8.9% (2.7%, 25.7%)	0.5% (0.1%, 2.8%)	22.20%
	30	56.3% (34.7%, 75.7%)	30% (15.8%, 49.4%)	2.1% (0.5%, 8.5%)	54.20%
	40	85% (62.1%, 95.1%)	65.3% (38.6%, 84.9%)	8.6% (1.9%, 31.8%)	76.40%
**75**	20	56% (28.9%, 80%)	29.8% (12%, 56.8%)	2.1% (0.5%, 8.9%)	53.90%
	30	84.8% (69.1%, 93.3%)	65% (46.3%, 80%)	8.5% (2.6%, 24.3%)	76.30%
	40	96.1% (86.5%, 98.9%)	89.1% (72.1%, 96.3%)	28.9% (8.5%, 64%)	67.20%
**80**	20	84.7% (62.7%, 94.8%)	64.8% (37.6%, 84.9%)	8.4% (2.4%, 25.8%)	76.30%
	30	96% (89.2%, 98.6%)	89% (77.1%, 95.1%)	28.7% (11.8%, 54.6%)	67.30%
	40	99.1% (95.8%, 99.8%)	97.2% (90.5%, 99.2%)	63.8% (29.6%, 88.1%)	35.30%
**85**	20	96% (86.3%, 98.9%)	88.9% (70.3%, 96.4%)	28.4% (10.5%, 57.4%)	67.60%
	30	99.1% (96.5%, 99.7%)	97.2% (92.1%, 99.1%)	63.6% (37.5%, 83.5%)	35.50%
	40	99.8% (98.7%, 100%)	99.4% (97%, 99.9%)	88.4% (63.4%, 97.1%)	11.40%
**90**	20	99% (95.6%, 99.8%)	97.2% (89.4%, 99.3%)	63.3% (33.8%, 85.4%)	35.70%
	30	99.8% (98.9%, 100%)	99.3% (97.4%, 99.8%)	88.3% (70.3%, 96%)	11.50%
	40	100% (99.6%, 100%)	99.8% (99.1%, 100%)	97.1% (86.7%, 99.4%)	2.90%
**95**	20	99.8% (98.6%, 100%)	99.3% (96.6%, 99.9%)	88.2% (66.6%, 96.6%)	11.60%
	30	99.9% (99.6%, 100%)	99.8% (99.2%, 100%)	97% (89.5%, 99.2%)	2.90%
	40	100% (99.9%, 100%)	100% (99.7%, 100%)	99.3% (95.9%, 99.9%)	0.70%
**100**	20	99.9% (99.6%, 100%)	99.8% (98.9%, 100%)	97% (87.8%, 99.3%)	2.90%
	30	100% (99.9%, 100%)	100% (99.7%, 100%)	99.3% (96.6%, 99.9%)	0.70%
	40	100% (100%, 100%)	100% (99.9%, 100%)	99.8% (98.7%, 100%)	0.20%
**105**	20	100% (99.9%, 100%)	100% (99.7%, 100%)	99.3% (96.1%, 99.9%)	0.70%
	30	100% (100%, 100%)	100% (99.9%, 100%)	99.8% (98.9%, 100%)	0.20%
	40	100% (100%, 100%)	100% (100%, 100%)	100% (99.6%, 100%)	0%
**110**	20	100% (100%, 100%)	100% (99.9%, 100%)	99.8% (98.8%, 100%)	0.20%
	30	100% (100%, 100%)	100% (100%, 100%)	100% (99.7%, 100%)	0%
	40	100% (100%, 100%)	100% (100%, 100%)	100% (99.9%, 100%)	0%
**115**	20	100% (100%, 100%)	100% (100%, 100%)	100% (99.6%, 100%)	0%
	30	100% (100%, 100%)	100% (100%, 100%)	100% (99.9%, 100%)	0%
	40	100% (100%, 100%)	100% (100%, 100%)	100% (100%, 100%)	0%
**120**	20	100% (100%, 100%)	100% (100%, 100%)	100% (99.9%, 100%)	0%
	30	100% (100%, 100%)	100% (100%, 100%)	100% (100%, 100%)	0%
	40	100% (100%, 100%)	100% (100%, 100%)	100% (100%, 100%)	0%

^a^The difference between the highest predicted risk (0 AGG interruptions) and the lowest predicted risk (2 or 3 AGG interruptions).

### Magnitude of instability during transmission of intermediate and premutation alleles

We analyzed 244 transmission events from 192 parent carriers (179 mothers and 13 fathers) of an intermediate or premutation allele that increased in size but did not expand to a full mutation. Additional file 3: Figure S2 shows the distribution of maternal allele CGG repeat sizes, grouped by number of AGG interruptions. Maternal and paternal transmissions were also analyzed together, but when combined the distribution was only marginally changed. Too few paternal transmissions were observed to analyze separately. It has previously been shown that the instability of the CGG repeat locus is higher in paternal than maternal transmissions in the normal and intermediate range [11], so in the subsequent analyses only maternal transmissions are reported.

**Figure 4:**
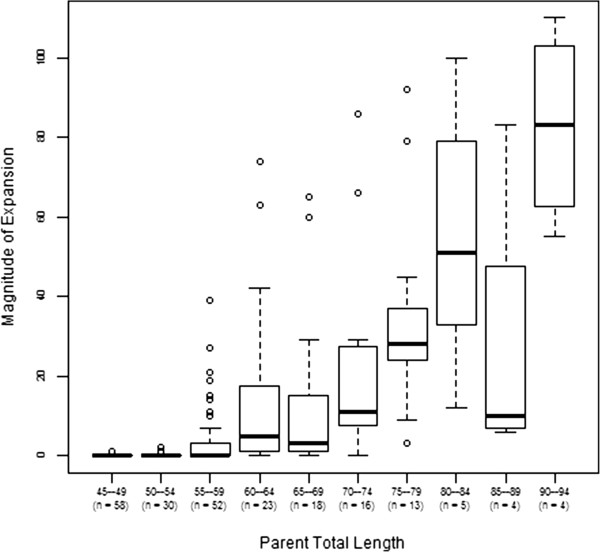
**Magnitude of instability of unstable alleles that do not expand to a full mutation.** Instability, as measured by the number of CGG repeats an allele increases during transmission, increases with an increase in CGG repeat size.

Table 2 shows the results of the mixed effects linear regression analysis of magnitude of expansion. The magnitude of expansion increased significantly with increasing total CGG length (*P* <0.001), with a predicted increase of 1.3 repeats in magnitude of expansion for each additional parental CGG repeat (Figure 4). The magnitude of expansion decreased significantly with increasing numbers of AGG interruptions, with an estimated difference of 9.8 fewer repeats in expansion with 1 parental AGG interruption *versus* 0 interruptions (*P* = 0.031), and an estimated difference of 15.2 fewer repeats in expansion with 2 or 3 AGG interruptions *versus* 0 interruptions (*P* = 0.001). Results are similar to those for both mothers and fathers.

**Table 2:** Mixed effects linear regression analysis of magnitude of expansion

**Covariate**	**Parameter estimate**^**a**^	**95% CI for parameter estimate**	***P*** **value**
Total CGG length	1.3	(1.0, 1.6)	<0.001
AGG (1 *vs*. 0 interruptions)	-9.8	(-18.8, -0.9)	0.031
AGG (2 or 3 *vs*. 0 interruptions)	-15.2	(-23.5, -6.8)	0.001
AGG (2 or 3 *vs*. 1 interruptions	-5.3	(-12.4, 1.8)	0.142
Parent age	0	(-0.4, 0.5)	0.919

^a^Incremental change in magnitude of expansion for each unit increase in a continuous covariate, or difference in magnitude of expansion between levels of a categorical covariate.

Using binomial logistic regression, the odds of instability of maternal transmissions (premutation and intermediate) were estimated to increase significantly for each additional maternal CGG repeat (*P* <0.001). The odds of instability decreased significantly with increasing numbers of AGG interruptions, (1 interruption *versus* 0 interruptions (*P* = 0.002), and 2 or 3 interruptions *versus* 0 interruptions (*P* <0.001).

### AGG distribution patterns

The distribution of AGG interruption patterns within 514 normal alleles from premutation mothers and from 797 normal alleles from females with two normal alleles differed significantly between the two groups (*P* <0.001). No significant difference was seen in the proportion of alleles with the pattern 10-A-9-A-9 (*P* = 0.713) but significantly fewer premutation subjects had the patterns 9-A-9-A-9 (*P* = 0.001) and 10-A-9-A-10 (*P* = 0.024) and significantly more premutation subjects had the pattern 10-A-9 (*P* = 0.001).

The total CGG length of normal alleles did not differ significantly between groups (*P* = 0.267), with both groups having a mean of 29.

## Discussion

### Risk of expansion to a full mutation in maternal premutation alleles

It has been demonstrated that the AGG interruption pattern in *FMR1* intermediate and premutation alleles can refine the risk of instability during parental transmission and it should be validated in genetic counseling prediction models [10,11]. To increase the utility of AGG interruption information to better predict the risk of expansion to a full mutation when counseling a premutation mother, we have updated our previous study that investigated the role of AGG interruptions in the stability of *FMR1* during maternal transmission.

A total of 710 transmissions from 525 premutation carrier mothers were considered in this study, approximately doubling the transmissions used in a previous study [10]. This collection includes transmissions from the UC Davis MIND Institute (135 from 102 mothers), the Hospital Sant Joan de Déu (105 from 84 mothers), the University of Padova (16 from 14 mothers), Rush University (86 from 61 mothers), and our previously published study (368 from 264 mothers). Thus, the updated cohort is both more extensive and more genetically diverse than our previous study, which included only participants who were recruited through the UC Davis MIND Institute.

Initially, we recalculated the risk of an allele expanding to a full mutation during maternal transmission using total CGG repeat length and number of AGG interruptions of the maternal premutation allele. In agreement with our previous report [10] alleles that had a total length of approximately 75 CGG repeats were determined to have the greatest difference in the frequency of expansion based on the number of AGG interruptions present. The model predicts that 83% (CI: 72.0%, 89.7%) of alleles with 75 total CGG repeats would expand to a full mutation when no AGG interruptions are present (75 CGG repeat pure stretch), but only 17% (CI: 8.2%, 31.7%) would expand to a full mutation when 2 or 3 AGG interruptions are present. All predicted risks are within the confidence intervals estimated in the previously published model. Alleles with 0, 1, 2 or 3 AGG interruptions are all estimated to expand to a full mutation in more than 90% of transmissions when the total length exceeds 95 CGG repeats.

Three of the 525 maternal premutation alleles had 3 AGG interruptions, we combined this group with alleles that were interrupted with 2 AGGs. It is likely that analyzing more transmissions of alleles with 3 AGG interruptions will show differences in the frequency of full mutation expansions when compared to alleles with 2 AGG interruptions. However, it has been shown in several studies that alleles with greater than 2 AGG interruptions are rare compared to the commonly observed patterns with 0, 1 or 2 AGG interruptions [4,11].

An increase in the odds of expansion to a full mutation was found to exist for each additional CGG repeat, but the odds of expansion decreased by 2.4- or 23-fold (243% or 2,434%) with 1, or 2 or 3 AGG interruptions, respectively. Additionally, a 16% increase in odds of expansion to a full mutation (defined as the probability of a full mutation child divided by the probability of a premutation child) occurred for every year of the mother’s age at the time of childbirth. While changes in the total length of the CGG repeat locus has the greatest affect on the risk of the allele expanding to a full mutation, our findings indicate an additive effect of AGG interruptions and maternal age, which appear to substantially influence the frequency with which expansions are expected.

The observation that maternal age is a risk factor for expansion to a full mutation during maternal transmission is an intriguing finding from this study. Previous studies have also examined maternal age as a variable associated with instability of the CGG repeat locus. Two studies did detect a correlation between age of the mother and the size of the CGG repeat. A sibling study found an increase in the mean size of the full mutation allele in the younger sibling [24], and a study of premutation carrier offspring observed an association between maternal age and mutation status (premutation or full mutation) of the offspring, however when samples retrospectively collected were analyzed separately from samples collected prospectively, the maternal age effect was only present for the retrospective study, suggesting that recruitment bias rather than a true age effect could be responsible for the findings [25]. No statistically significant association of maternal age upon the risk of expansion to a full mutation was found in two more recent studies that incorporated AGG interruptions into the analysis although the sample size included was much smaller than the one presented here [10,11]. As a result, additional data are warranted to further explore the risk associated with maternal age. If supported by further studies, then maternal age would be the first risk factor for CGG repeat expansion that an individual can use to lower their risk of having a child with FXS. This knowledge would also provide an increased incentive for individuals to be screened for a premutation allele at an early age.

The observed maternal age effect in our cohort could be explained by ascertainment bias; having a child with a full mutation may influence reproductive behavior, causing the women in our sample to delay or continue child-bearing until older ages. The age of mothers with premutation children was very similar to mothers with full mutation children, however further analyses should be performed on prospective cohorts in order to verify the role played by maternal age in risk of expansion to a full mutation.

The observed data for three separated collection sites (UC Davis, Rush University, and Hospital Sant Joan de Dèu), which included 135, 86, and 105 samples, showed a greater fluctuation in the frequency of alleles expanding to a full mutation due to a small sample size. However, the same key features were present for each cohort; the bivariable risk model (total CGG length and number of AGG interruptions) consistently predicted an increase in risk of expansion of the allele to a full mutation when total length increased in size. The observed similarities of predicted risks between cohorts suggest that the usage of a single model in a genetic counseling setting will be beneficial across various ethnicities; however more studies are warranted to further confirm these findings.

A model that calculates risk of expansion to a full mutation using pure CGG stretch, number of AGG interruptions, and maternal age produced the lowest AIC score of 235.6. Indeed, when pure CGG stretch is replaced with total CGG length the model has a higher AIC score of 241.6. However, while the model that incorporates pure CGG stretch is nominally the best fit model using the AIC, it predicts the highest risk of expansion to occur if an allele has 1 AGG interruption compared to having 0, 2, or 3 AGG interruptions, an inconsistency that is likely the result of an insufficient number of observations in the group with 2 or 3 AGG interruptions. A revised risk model calculated using logistic regression of the 710 maternal transmissions, considered the total length of the CGG repeat allele, the number of AGG interruptions, and the age of the mother at childbirth. This model was determined to be more suitable than that which considered pure CGG stretch instead of total length, confirming the previous data on a smaller sample [10].

### Instability of intermediate and premutation alleles

Previous studies have shown significant differences in the frequency of instabilities between male and female parents [11]; however we did not observe a significant difference likely due to the small sample size of paternal transmissions included.

The odds of instability increased significantly with CGG repeat length (*P* <0.001) and decreased significantly with number of AGG interruptions (*P* = 0.002) in the parental allele, consistent with previous reports [11].

Magnitude of expansion was significantly correlated with total CGG repeat length in our maternal cohort and was significantly reduced when more AGG interruptions were present in the maternal allele, these findings are consistent with Nolin et al. [11] where the presence of AGG interruptions was shown to decrease the magnitude of instability. Inclusion of paternal transmissions had only a minimal effect on the outcomes of the above results.

When parental age was tested to determine if it could significantly predict either the frequency of instability (excluding expansions to full mutations) or the magnitude of instability, no effect was found for either maternal (*P* = 0.919, instability; *P* = 0.774, magnitude) or maternal and paternal transmissions (*P* = 0.892, instability; *P* = 0.907, magnitude).

## Conclusions

The results of this study demonstrate the value that can be gained by incorporating information about AGG interruptions when estimating the instability of an intermediate or premutation allele following transmission. It is clear that the CGG repeat element is more stable when interrupted by AGG triplets, and this is true for both intermediate and premutation alleles. The presence of AGG interruptions affects how likely it is that an allele is unstable, how often it will expand to a full mutation, and the magnitude of expansion during transmission. While the length of the CGG allele (total or pure stretch) is the single best variable that predicts the stability of the allele during transmission, information about AGG interruptions and maternal age can significantly increase the accuracy of risk assessments, providing more complete information for premutation carriers and individuals with an intermediate allele during genetic counseling. Thus, we consider a model that incorporates total length of the CGG allele, number of AGG interruptions, and maternal age to be the best model for predicting the risk of expansion to a full mutation. In some cases, the additional information obtained from AGG analysis may represent a key factor in reproductive decision-making for carriers. Knowledge gained from AGG analysis may impact parental decisions to conceive a pregnancy naturally, pursue assisted reproductive technologies, such as preimplantation genetic diagnosis, or utilize prenatal diagnostic procedures. Pre- and post-testing genetic counseling is a necessary tool in assisting patients to understand the impact of AGG analysis on their risk assessment and reproductive options. Although no systematic investigations have been done to look at the clinical utility of AGG analysis, anecdotal experience have shown that for patients with 60 to 80 CGG repeats, knowledge added by AGG analysis has impacted their reproductive decisions. Although the ultimate reproductive decision may not change with the AGG information, it seems that premutation women, who have undergone AGG analysis, feel more reassured, have a more clear perception of their risk, and can make a more confident choice. Future studies are needed to investigate the clinical implications of AGG analysis on patient reproductive decision-making in a systematic way. Such studies should not only take into account reproductive outcomes but should also look at changes in risk perception, anxiety, and perceived value related to AGG analysis.

While maternal age appears to play a role in the risk of expansion to a full mutation, interestingly we did not observe the same to be true for instability of the allele (any increase in CGG repeat size when an intermediate or small premutation allele), perhaps because a smaller sample size was analyzed when considering instability. Previously, studies have been inconclusive or did not observe a maternal age affect on the expansion of a premutation allele to a full mutation during maternal transmission, however both maternal and paternal age are known to contribute to the risk of trinucleotide repeat expansion in other disorders [10,24-28].

Lastly, the information in this study and in recent publications can be used by genetic counselors to provide a better understanding of the risk of the CGG repeat allele transmitting unstably, or expanding to a full mutation, and provides better tools for informed decision-making, particularly before a pregnancy, in female carriers of a premutation allele. The AGG information can be obtained using a PCR-based assay, which can also provide CGG allele size information. However, the test has to be separately ordered as it is not routinely part of carrier testing.

## Competing interests

CMY, LM, BDJ, MN, JG, AM, RP, LZ, DB, AR, and BF have no disclosures. FT has received funds from Roche. EBK has received support from Asuragen, Inc. in the development of control samples for *FMR1* testing. GJL and AH have stock options in Asuragen, Inc.

## Authors’ contributions

CMY contributed to the design experiments, performed experiments, helped with data analysis, and prepared the manuscript. LM, AM, and EBK provided samples, provided critical review of the manuscript. BDJ performed all statistical analyses and provided critical review of the manuscript. MN, JG, RP, and LZ performed experiments and provided critical review of the manuscript. DB, ARD, and BF contributed genetic counseling related text to the manuscript and provided critical review of the manuscript. GL and AH provided critical review of the manuscript. FT conceived of the study, provided samples, designed the experiments, and prepared the manuscript. All authors have read and approved the final manuscript.

## Supplementary Material

Additional file 1: Table S1 Participants included in the analysis. **Table S2.** Predicted risk of expansion to a full mutation using total CGG length and AGGs. **Table S3.** Summary of 710 observed transmissions from premutation carrier mothers. **Table S4.** Binomial logistic regression analysis of instability in premutation mothers. Click here for file

Additional file 2: Figure S1 Percent of transmissions of maternal premutation alleles that resulted in a full mutation child. The observed frequency of children with a full mutation grouped by 0 (black line), 1 (red line), and 2 or 3 (green line) AGG interruptions in the maternal premutation allele increases with increased CGG size and decreases with increased number of AGG interruptions. Data were corrected for mothers with multiple children. Click here for file

Additional file 3: Figure S2 Instability measures of maternal intermediate and premutation alleles. Instability of the CGG repeat allele increases with the total length of the allele. The proportion of alleles with 0 (black), 1 (red), and 2 or 3 (green) AGG interruptions, that are unstable, changes as alleles become more unstable and begin expanding to a full mutation (0 and 1 AGG interruptions). A higher proportion of alleles with 2 or 3 AGG interruptions are observed for longer repeats as they do not expand to a full mutation. Click here for file
