# Supplementary material for: AGG interruptions and maternal age affect FMR1 CGG repeat allele stability during transmission
Source: J Neurodev Disord. 2014 Jul 30;6(1):24. doi: 10.1186/1866-1955-6-24 (PMC4126815; doi:10.1186/1866-1955-6-24)
Supplement: Additional file 1: Table S1 — Participants included in the analysis. Table S2. Predicted risk of expansion to a full mutation using total CGG length and AGGs. Table S3. Summary of 710 observed transmissions from premutation carrier mothers. Table S4. Binomial logistic regression analysis of instability in premutation mothers. [file 1866-1955-6-24-S1.docx]

**Supplementary Table 1 Participants included in the analysis**

| **Analysis** | **Parent** | **Parental Category** | **Number of Participants** | **Transmissions/**  **Observed Child Category** |
| --- | --- | --- | --- | --- |
| Magnitude of expansion within the premutation range | Fathers | Intermediate | 13 | 14 Intermediate / 6 Premutation |
|  | Mothers | Intermediate | 62 | 86 Intermediate / 2 Premutation |
|  |  | Premutation | 117^a^ | 136^b^ Premutation |
| Risk of expansion to a full mutation | Mothers | Premutation | 525 | 166 Premutation / 544 Full Mutation |

| ^a^ Number of premutation carrier mothers (out of 525) that had children with a premutation allele where total CGG length data was known. |
| --- |
| ^b^ Number of transmissions where the child's allele was a premutation allele of known size.  **Supplementary Table 2 Predicted risk of expansion to a full mutation using total CGG length and AGGs** |

| Total length | 0 AGG interruptions | 1 AGG interruption | 2 or 3 AGG interruptions | Differential Risk |
| --- | --- | --- | --- | --- |
| 55 | 5.6%(2.4%, 12.4%) | 2.4%(0.9%, 6.1%) | 0.3%(0.1%, 1%) | 5.30% |
| 60 | 15.1%(7.9%, 26.9%) | 6.8%(3.1%, 14.1%) | 0.8%(0.2%, 2.6%) | 14.30% |
| 65 | 34.6%(22.1%, 49.7%) | 17.9%(10%, 30%) | 2.2%(0.8%, 6.3%) | 32.40% |
| 70 | 61.3%(47%, 73.9%) | 39.4%(26.7%, 53.8%) | 6.4%(2.6%, 14.8%) | 54.90% |
| 75 | 82.5%(72%, 89.7%) | 66%(52.8%, 77.2%) | 16.9%(8.2%, 31.7%) | 65.60% |
| 80 | 93.4%(87.5%, 96.6%) | 85.3%(76.1%, 91.4%) | 37.8%(22.1%, 56.5%) | 55.60% |
| 85 | 97.7%(94.8%, 99%) | 94.5%(89.5%, 97.2%) | 64.4%(46.1%, 79.3%) | 33.30% |
| 90 | 99.2%(97.9%, 99.7%) | 98.1%(95.6%, 99.2%) | 84.4%(71%, 92.3%) | 14.80% |
| 95 | 99.7%(99.1%, 99.9%) | 99.4%(98.2%, 99.8%) | 94.2%(86.9%, 97.5%) | 5.50% |
| 100 | 99.9%(99.7%, 100%) | 99.8%(99.3%, 99.9%) | 98%(94.6%, 99.3%) | 1.90% |
| 105 | 100%(99.9%, 100%) | 99.9%(99.7%, 100%) | 99.3%(97.8%, 99.8%) | 0.70% |
| 110 | 100%(99.9%, 100%) | 100%(99.9%, 100%) | 99.8%(99.1%, 99.9%) | 0.20% |
| 115 | 100%(100%, 100%) | 100%(100%, 100%) | 99.9%(99.6%, 100%) | 0.10% |
| 120 | 100%(100%, 100%) | 100%(100%, 100%) | 100%(99.9%, 100%) | 0% |

^a^ The difference between the highest predicted risk (0 AGG interruptions) and the lowest predicted risk (2 or 3 AGG interruptions).

**Supplementary Table 3 Summary of 710 observed transmissions from premutation carrier mothers**

| Maternal Total Length | Total | No. With Premutations | No. With Full Mutation | No. With 0 AGGs | No. With 1 AGGs | No. With 2 AGGs | No. With 3 AGGs |
| --- | --- | --- | --- | --- | --- | --- | --- |
| 50-59 | 56 | 54 | 2 | 11 | 9 | 34 | 2 |
| 60-69 | 60 | 54 | 6 | 25 | 16 | 19 | 0 |
| 70-79 | 135 | 38 | 97 | 77 | 51 | 7 | 0 |
| 80-89 | 154 | 13 | 141 | 66 | 61 | 27 | 0 |
| 90-99 | 107 | 6 | 101 | 39 | 42 | 26 | 0 |
| 100-109 | 100 | 1 | 99 | 57 | 24 | 19 | 0 |
| 110-119 | 30 | 0 | 30 | 21 | 6 | 3 | 0 |
| 120-129 | 38 | 0 | 38 | 32 | 4 | 2 | 0 |
| 130-139 | 15 | 0 | 15 | 10 | 2 | 3 | 0 |
| 140-149 | 6 | 0 | 6 | 5 | 1 | 0 | 0 |
| 150-159 | 3 | 0 | 3 | 3 | 0 | 0 | 0 |
| 160-169 | 2 | 0 | 2 | 2 | 0 | 0 | 0 |
| 170-179 | 2 | 0 | 2 | 2 | 0 | 0 | 0 |

| **Supplementary Table 4 Binomial logistic regression analysis of instability in premutation mothers** | | | |
| --- | --- | --- | --- |
| **Covariate** | **Odds Ratio** | **95% CI for Odds Ratio** | **P-Value** |
| Total CGG Length | 1.29 | (1.19, 1.42) | <0.001 |
| AGG (1 vs. 0 Interruptions) | 0.07 | (0.01, 0.43) | 0.008 |
| AGG (2 or 3 vs. 0 Interruptions) | 0.01 | (0.00, 0.07) | <0.001 |
| Parent Age | 0.98 | (0.87, 1.11) | 0.801 |

**Supplemental Figure 1 Percent of transmissions of maternal premutation alleles that resulted in a full mutation child.** The observed frequency of children with a full mutation grouped by 0 (black line), 1 (red line), and 2 or 3 (green line) AGG interruptions in the maternal premutation allele increases with increased CGG size and decreases with increased number of AGG interruptions. Data were corrected for mothers with multiple children.

**Supplementary Figure 2 instability measures of maternal intermediate and premutation alleles.** Instability of the CGG repeat allele increases with the total length of the allele. The proportion of alleles with 0 (black), 1 (red), and 2 or 3 (green) AGG interruptions, that are unstable, changes as alleles become more unstable and begin expanding to a full mutation (0 and 1 AGG interruptions). A higher proportion of alleles with 2 or 3 AGG interruptions are observed for longer repeats as they do not expand to a full mutation.
