# Supplementary figures and images for: AGG interruptions and maternal age affect FMR1 CGG repeat allele stability during transmission
Source: J Neurodev Disord. 2014 Jul 30;6(1):24. doi: 10.1186/1866-1955-6-24 (PMC4126815; doi:10.1186/1866-1955-6-24)

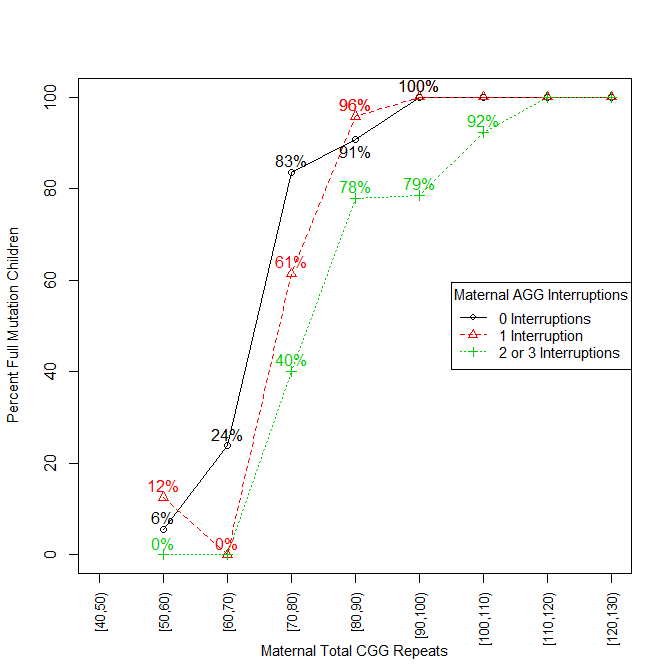

Supplement: Additional file 2: Figure S1 — Percent of transmissions of maternal premutation alleles that resulted in a full mutation child. The observed frequency of children with a full mutation grouped by 0 (black line), 1 (red line), and 2 or 3 (green line) AGG interruptions in the maternal premutation allele increases with increased CGG size and decreases with increased number of AGG interruptions. Data were corrected for mothers with multiple children. [file 1866-1955-6-24-S2.tiff]

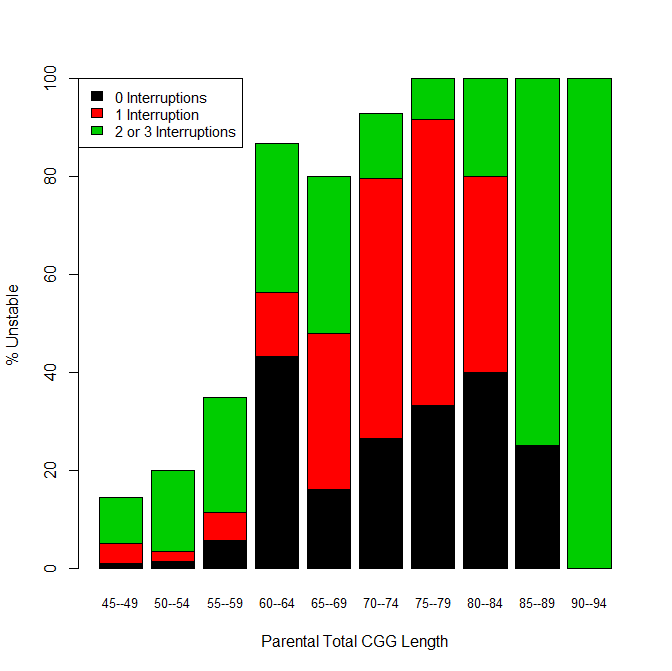

Supplement: Additional file 3: Figure S2 — Instability measures of maternal intermediate and premutation alleles. Instability of the CGG repeat allele increases with the total length of the allele. The proportion of alleles with 0 (black), 1 (red), and 2 or 3 (green) AGG interruptions, that are unstable, changes as alleles become more unstable and begin expanding to a full mutation (0 and 1 AGG interruptions). A higher proportion of alleles with 2 or 3 AGG interruptions are observed for longer repeats as they do not expand to a full mutation. [file 1866-1955-6-24-S3.tiff]
